# *Lactobacillus helveticus* SBT2171 Induces A20 Expression via Toll-Like Receptor 2 Signaling and Inhibits the Lipopolysaccharide-Induced Activation of Nuclear Factor-kappa B and Mitogen-Activated Protein Kinases in Peritoneal Macrophages

**DOI:** 10.3389/fimmu.2019.00845

**Published:** 2019-04-17

**Authors:** Michio Kawano, Masaya Miyoshi, Tadaaki Miyazaki

**Affiliations:** ^1^Milk Science Research Institute, Megmilk Snow Brand Co. Ltd., Saitama, Japan; ^2^Department of Probiotics Immunology, Institute for Genetic Medicine, Hokkaido University, Hokkaido, Japan

**Keywords:** lactic acid bacteria, *Lactobacillus helveticus* SBT2171, cytokine production, antigen-presenting cell, nuclear factor-kappa B, mitogen-activated protein kinase, A20, toll-like receptor 2

## Abstract

*Lactobacillus helveticus* SBT2171 (LH2171) has been reported to ameliorate the development of autoimmune diseases, such as collagen-induced arthritis and experimental autoimmune encephalitis in mice and inhibit interleukin (IL)-6 production in antigen-presenting cells *in vitro*. Regulation of cytokine production by antigen-presenting cells might be critical for the anti-inflammatory function of LH2171 in autoimmune diseases. However, the mechanism and contributing components of LH2171-mediated inhibition of IL-6 production are unclear. Here, we examined the anti-inflammatory effects of LH2171 in lipopolysaccharide (LPS)-stimulated peritoneal macrophages, as a model of antigen-presenting cells, necessary for the pathogenesis of autoimmune diseases. LH2171 significantly reduced LPS-induced expression and secretion of IL-6 and IL-1β cytokines. It also inhibited activation of nuclear factor-kappa B and mitogen-activated protein kinases (NF-κB/MAPKs). Moreover, LH2171 induced gene expression of several negative regulators of NF-κB/MAPKs. Among these regulators, A20 was strongly up-regulated at the mRNA and protein levels upon LH2171 treatment. The cell wall fraction of LH2171 also demonstrated a similar increase in A20 gene expression and exerted an anti-inflammatory effect. These results suggest that the cell wall may be one of the anti-inflammatory components of LH2171. Since cell wall components of Gram-positive bacteria are recognized by toll-like receptor 2 (TLR2), we investigated whether the anti-inflammatory effect of LH2171 was mediated by TLR2 signaling. Specifically, LH2171-mediated IL-6 suppression and A20 upregulation in wild-type macrophages were reversed and significantly reduced in TLR2 knock-out macrophages. These results suggest that LH2171 induces A20 expression via TLR2 signaling, inhibiting the activation of NF-κB/MAPKs and cytokine production in antigen-presenting cells. This might contribute to the anti-inflammatory activity of LH2171 on autoimmune diseases.

## Introduction

Regulation of immune responses is important for the prevention of many diseases. Activation of an immune response is required to eliminate virus-infected cells or cancer cells, whereas an excessive inflammatory response may result in the onset of a physiological disorder or even death. Therefore, the inflammatory response is precisely regulated in healthy individuals. Excessive inflammation is caused by stimulation of pathogen-associated molecular patterns (PAMPs) via toll-like receptors (TLRs) (1). One of the most famous PAMPs, lipopolysaccharide (LPS), which is a Gram-negative bacterial component, is recognized by TLR4 (2). LPS activates inflammatory signaling pathways, which are mediated by molecules, such as nuclear factor-kappa B (NF-κB) and mitogen-activated protein kinases (MAPKs) (2). In the last two decades, it has been proven that TLR signaling could be activated by PAMPs and damage-associated molecular patterns (DAMPs), which are endogenous danger signals produced by injured or dead cells (3–5). Recent studies indicate that activation of TLR signaling pathways by PAMPs or DAMPs has an essential role in the pathogenesis and development of inflammatory diseases, such as inflammatory bowel disease, rheumatoid arthritis, and multiple sclerosis (6–9). Indeed, it was reported that the severity of murine collagen-induced arthritis (CIA), a model of human rheumatoid arthritis, was suppressed upon administration of a TLR4 antagonist (10).

Lactic acid bacteria (LAB) are beneficial microorganisms widely used in the manufacturing of various fermented foods. LAB are known to modulate immune responses (11). Some reports have shown that LAB consumption activates innate immunity and induces host anti-viral responses (12), while other reports have demonstrated that LAB ameliorate excessive inflammation (13). Immune modulation as a result of LAB administration includes the regulation of TLR signals in immune cells. For instance, *Lactococcus lactis* JCM5805 activates TLR9 signaling in plasmacytoid dendritic cells and induces interferon-α production, which exerts anti-viral capacity (14). *Tetragenococcus halophilus* KK221, which is one of the LAB used to manufacture soy sauce, stimulates dendritic cells via TLR3 to produce interferon-β and inhibits colonic inflammation in mice with dextran sodium sulfate-induced colitis (15). A strain of *Lactobacillus* (*L*.) *plantarum* has been shown to induce IL-10 production via TLR2-dependent pathways and prevent IL-12 production in macrophages (16). These studies suggested that regulation of TLR signaling is important for the beneficial effects of LAB against viral infection or inflammatory disease.

*L. helveticus* SBT2171 (LH2171) is one of the LAB used in Gouda cheese production. It has been reported to have anti-inflammatory properties. An *in vitro* study has shown that LH2171 prevents proliferation and cytokine production in LPS-stimulated murine splenocytes (17). Oral administration of LH2171 in mice with CIA showed a reduction in joint swelling and serum levels of anti-bovine type II collagen antibody (18). Additionally, these preventive effects on CIA symptoms and inflammatory responses were more clearly observed in mice intraperitoneally injected with heat-killed LH2171 (18, 19). Intraperitoneal injection of heat-killed LH2171 in mice also prevented inflammatory responses and pathogenesis of experimental autoimmune encephalomyelitis (EAE), a model of multiple sclerosis (20). These observations suggested that LH2171 itself, rather than LH2171-produced metabolites in the intestines, suppressed inflammatory responses in inflammatory diseases. Furthermore, a previous study has shown that LH2171 prevents IL-6 production in LPS-stimulated antigen-presenting cells (20). IL-6 is one of the most important pro-inflammatory cytokines responsible for the pathogenesis and development of CIA and EAE (21). Therefore, it was suggested that inhibition of IL-6 production in antigen-presenting cells might contribute to the inhibitory effects of LH2171 cells on systemic inflammation in these inflammatory diseases. However, the mechanism of LH2171-mediated inhibition of IL-6 production in antigen-presenting cells and the components involved remain to be elucidated.

In the present study, peritoneal macrophages were used as a model of antigen-presenting cells to examine the effects of LH2171. We investigated LH2171-mediated suppression of both pro-inflammatory cytokine production and intracellular signal activation by TLR4 agonist, LPS. Subsequently, we focused on the regulatory effect of LH2171 on TLR signaling pathways controlled by negative regulators. Furthermore, we investigated the component of LH2171 which was responsible for the anti-inflammatory effect on peritoneal macrophages.

## Materials and Methods

### Materials

Lipopolysaccharide (LPS) derived from *E. coli* 055:B5 and penicillin-streptomycin solution were purchased from Sigma Aldrich (St. Louis, MO, USA). RPMI-1640 medium was purchased from Wako (Osaka, Japan). Fetal bovine serum (FBS), de Man, Rogosa, and Sharpe (MRS) broth, and Brewer's modified thioglycollate medium were purchased from BD Bioscience (Franklin Lakes, NJ, USA). Nigericin sodium salt was obtained from Enzo Life Sciences (Farmingdale, NY, USA). DNase I and pronase were purchased from Roche Diagnostics (Basel, Switzerland). RNase A was purchased from Nacalai Tesque (Kyoto, Japan).

### Preparation of Bacterial Cells

*L. helveticus* SBT2171 (LH2171) is a bacterial strain deposited in the International Patent Organism Depository, National Institute of Advanced Industrial Science and Technology (FERM BP−5445, Tsukuba, Ibaraki, Japan). *L. helveticus* JCM1120^T^ (LH1120^T^), *L. paracasei* ssp. *paracasei* JCM1149^T^ (LPA1149^T^), *L. plantarum* ssp. *plantarum* JCM8130^T^ (LPL8130^T^), and *L. acidophilus* JCM1132^T^ (LA1132^T^) were obtained from the Japan Collection of Microorganisms, RIKEN BioResource Center (Tsukuba, Ibaraki, Japan). Bacterial cells were cultured in sterile MRS broth at 37°C for 16 h. After cultivation, bacterial cells were collected by centrifugation (3,860 × *g*, 4°C, 10 min), washed twice with normal saline, and once with sterile water. These cells were lyophilized and resuspended in phosphate buffered saline (PBS) to make 10 mg/mL bacterial cell stocks. These cell stocks were heated at 80°C for 30 min, cooled on ice, and stored at −20°C until further use.

### Fractionation of LH2171 Cell Components

Preparation of LH2171 cell wall (CW) and peptidoglycan (PGN) was performed as described previously (16) with some modifications. Briefly, heat-killed LH2171 cells were resuspended in PBS (10 mg/mL) and disrupted three times using a French pressure cell (SLM Instruments, Urbana, IL, USA) at 1,200 psiG. Suspension of disrupted cells was centrifuged for 10 min at 10,400 × *g* and 4°C to separate the supernatant and precipitate. The supernatant was collected and filtered through a 0.2-μm syringe filter. The precipitate was delipidated through sequential suspension and centrifugation in methanol-water (1:1, v/v), methanol-chloroform-water (1:1:1, v/v), and methanol-chloroform (1:1, v/v). The delipidated precipitate was completely air-dried in a fume hood and then treated with 2.5 μg/mL DNase I and 2.5 μg/mL RNase A in 50 mM Tris-HCl (pH 7.5) for 16 h at 37°C. After 16 h, the preparation was washed once with sterile water and treated with 0.1 mg/mL pronase in 50 mM Tris-HCl (pH 7.5) for 24 h at 37°C. The precipitate treated with nuclease and protease was defined as the CW. Precipitate, delipidated precipitate, and CW were washed three times, lyophilized, and weighed. To prepare PGN, the CW was treated with 47% hydrofluoric acid at 4°C for 20 h. The material was washed three times, lyophilized, weighed, and then used as the PGN. The LH2171 cell components were stored at −20°C until use.

### Mice

C57BL/6J mice were purchased from Japan SLC Inc. (Shizuoka, Japan). TLR2 knock-out (KO) mice (C57BL/6J background) were purchased from Oriental Bio Service Inc. (Kyoto, Japan). Mice were housed in plastic cages in an air-conditioned and specific pathogen-free room (22–26°C, light exposure from 8:00 to 21:00) with free access to sterilized food and water. All experiments were carried out in accordance with the guidelines of the Bioscience Committee of Hokkaido University (Sapporo, Hokkaido, Japan) and were approved by the Animal Care and Use Committee of Hokkaido University.

### Preparation of Peritoneal Macrophages

Male mice (2–4 mice, 8–16 weeks old) were intraperitoneally injected with 2 mL sterilized 4% Brewer's modified thioglycollate medium. Three days after thioglycollate injection, these mice were deeply anesthetized and intraperitoneally injected with 5 mL PBS. After a gentle massage of the peritoneum, the PBS was collected and centrifuged to obtain the peritoneal exudate cells. These cells were suspended in cell culture medium (RPMI-1640 containing 10% FBS, 100 U/mL of penicillin, and 100 μg/mL streptomycin) and seeded in a 6-well plate (2 × 10^6^ cells/well), 12-well plate (1 × 10^6^ cells/well), or 96-well plate (1 × 10^5^ cells/well). Three hours after seeding, cells were washed once with PBS to remove floating cells. Adherent cells on these plates were cultured for 20 h and used as peritoneal macrophages for further experiments. The ratio of F4/80 positive cells per adherent cells, which were defined as peritoneal macrophages, was ~70% (Supplementary Figure 1).

Peritoneal macrophages were cultured with or without heat-killed *Lactobacilli* (1–50 μg/mL) or bacterial components (10 μg/mL) in culture medium for 4 h. Subsequently, LPS was added at a final concentration of 1 μg/mL. After LPS addition, peritoneal macrophages were further incubated for 16 h, unless otherwise indicated. To measure IL-1β levels in the culture supernatant, LPS-stimulated macrophages were additionally treated with 5 μM nigericin for 45 min.

### Cell Proliferation and Viability Assays

Cell proliferation and viability assays were performed on peritoneal macrophages cultured in the 96-well plate. Cell proliferation rate was measured using the Cell Counting Kit-8 (Dojindo, Kumamoto, Japan). Cell viability was measured by the amount of lactate dehydrogenase (LDH) activity released using the Cytotoxicity Detection Kit^PLUS^ (Roche Diagnostics). These assays were conducted according to the manufacturer's protocol.

### Cytokine Production Assay

Cytokine production assay was performed on peritoneal macrophages cultured in the 96-well plate. The culture medium was collected and centrifuged to remove debris. The supernatant was stored at −80°C until use. The concentrations of IL-1β and IL-6 in the cell culture supernatant were determined using commercial mouse ELISA MAX™ Kits (Biolegend, San Diego, CA, USA).

### Gene Expression Analysis

Peritoneal macrophages cultured in the 12-well plate were washed once with ice-cold PBS, and total RNA was isolated using the TRIzol reagent (Thermo Fisher Scientific, Waltham, MA, USA). Obtained RNA was reverse-transcribed into cDNA using the ReverTra Ace kit (Toyobo, Osaka, Japan). The real-time quantitative PCR (qPCR) assay was performed using the KAPA SYBR FAST Universal qPCR Kit (Kapa Biosystems, Boston, MA, USA) and StepOnePlus™ Real-Time PCR System (Thermo Fisher Scientific). RNA extraction, cDNA synthesis, and qPCR assay were performed according to the manufacturer's protocols. The relative levels of gene expression were calculated using the delta-delta Ct method. *Rpl19* was used as an endogenous control. Primer sequences are listed in Supplementary Table 1.

### Fluorescent Immunostaining

Peritoneal macrophages cultured on glass coverslips in a 6-well plate were washed once with ice-cold PBS and fixed with 4% paraformaldehyde in phosphate buffer (Nacalai Tesque) for 15 min at room temperature (RT, 20–26°C). Fixed cells were washed three times with PBS and permeabilized with 0.5% Triton X-100 in PBS for 15 min at RT. Subsequently, cells were washed three times with PBS and blocked with 2% BSA in PBS for 1 h at RT. The cells were stained with the anti-NF-κB p65 antibody listed in Supplementary Table 2, and the cell nuclei were stained with DAPI. The cells on glass coverslips were analyzed by confocal fluorescent microscopy (FV1000-D, Olympus, Tokyo, Japan).

### Western Blot Analysis

Peritoneal macrophages cultured in the 6-well plate were washed once with ice-cold PBS and lysed in RIPA buffer (10% glycerol, 50 mM Tris-HCl [pH 7.4], 150 mM NaCl, 1% NP-40, 0.5% sodium deoxycholate, 0.1% SDS, 1 × protease inhibitor cocktail [cOmplete mini, Roche], and 1 × phosphatase inhibitor cocktail [PhosSTOP, Roche]). The lysed cells were centrifuged to remove debris, and the supernatants were collected. The supernatants were adjusted to equal protein concentrations and mixed with an equal volume of 2 × sample buffer (10% 2-mercaptoethanol, 125 mM Tris-HCl [pH 6.8], 4% SDS, 10% sucrose, and 0.01% bromophenol blue). The mixed samples were heated at 95°C for 10 min, separated through 12.5% SDS-polyacrylamide gel electrophoresis (SDS-PAGE), and immunoblotted to detect proteins listed in Supplementary Table 2. Immunoblot images were captured using the LAS 4000 imaging system (GE Healthcare, Munich, Germany), and the band levels were quantified using the Image J software (National Institutes of Health, Bethesda, MD, USA).

### Statistical Analysis

Data were expressed as mean (either + or ±) SD. Statistical analyses were performed using the StatView software package 5.0 (SAS Institute, Inc.). For two-group comparisons, data were analyzed by Student's *t*-test. For multi-group comparisons, data were analyzed by Tukey-Kramer's test or Dunnett's test, which compared the LPS (or LPS + nigericin)-treated group with the other groups. *P*-values < 0.05 were considered statistically significant.

## Results

### LH2171 Inhibits Inflammatory Cytokine Production in LPS-Stimulated Peritoneal Macrophages

In a previous study, LH2171 was reported to suppress proliferation of primary immune cells isolated from murine mesenteric lymph nodes, Peyer's patches, and spleens (17). We first investigated whether LH2171 could exert a similar antiproliferative effect on peritoneal macrophages. LPS-stimulation of peritoneal macrophages significantly increased proliferation (Supplementary Figure 2A). Further, proliferation of LPS-stimulated macrophages was not affected by LH2171 pre-treatment at a dose of 1 and 10 μg/mL. However, pre-treatment with 50 μg/mL LH2171 strongly decreased the proliferation rate of LPS-stimulated macrophages to the level of the un-stimulated control (Supplementary Figure 2A). We also assessed the cytotoxic effect of LH2171 on peritoneal macrophages. LH2171 at 50 μg/mL induced 30% cytotoxicity, which did not occur at 1 and 10 μg/mL (Supplementary Figure 2B). Therefore, in LPS-stimulated peritoneal macrophages, LH2171 at a dose of 10 μg/mL or less did not affect cell proliferation or cell death.

**Figure 1:**
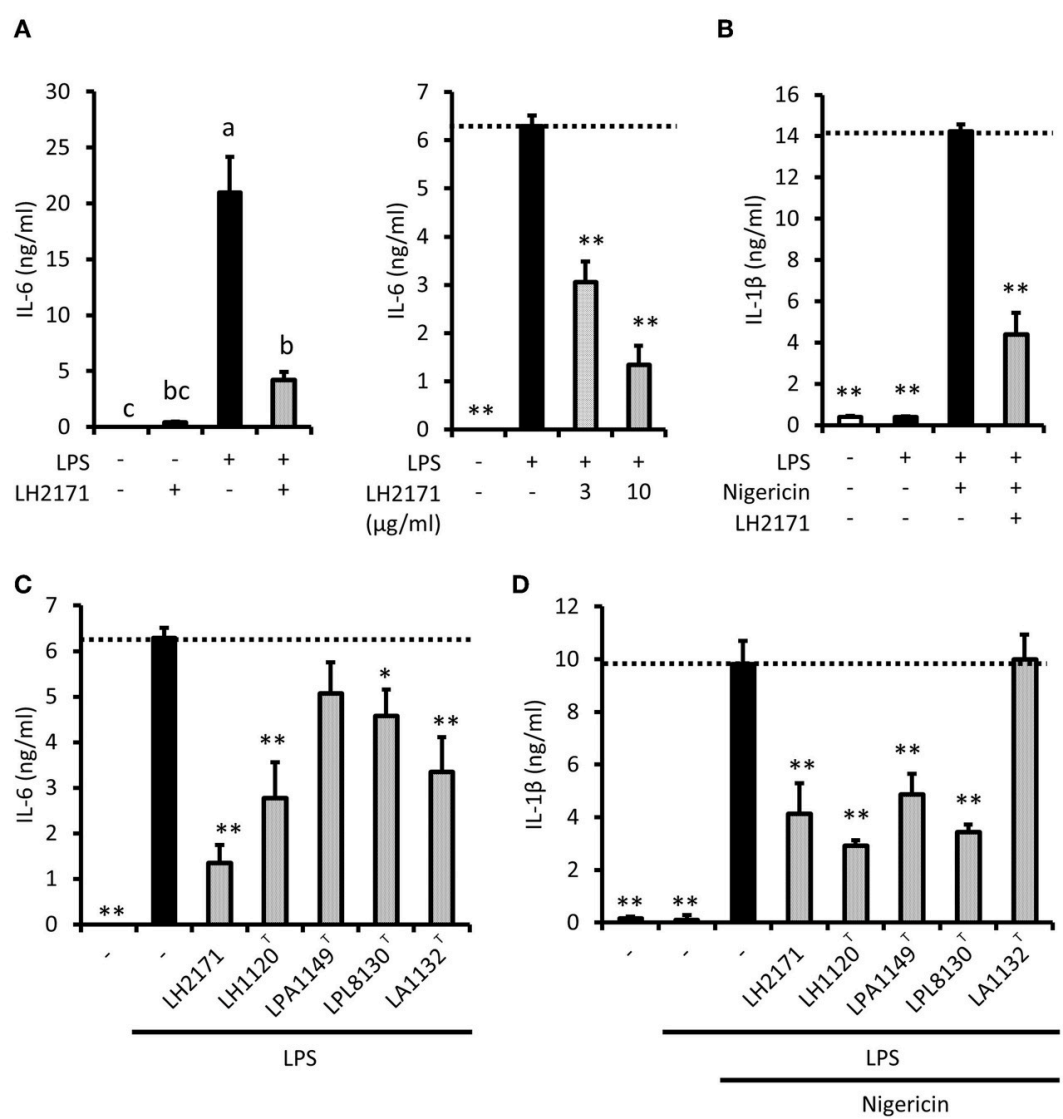
LH2171 inhibits production of inflammatory cytokines in LPS-stimulated peritoneal macrophages. The peritoneal macrophages were pre-incubated with or without LH2171 for 4 h and subsequently stimulated with 1 μg/mL LPS for 16 h. The levels of IL-6 in the supernatant was determined by ELISA **(A)**. LPS-stimulated macrophages were additionally treated with 5 μM nigericin for 45 min to measure IL-1β level in the cell culture supernatant **(B)**. The peritoneal macrophages were pre-incubated with different lactic acid bacteria for 4 h and stimulated with 1 μg/mL LPS for 16 h, and subsequently, IL-6 level in the culture supernatant was measured **(C)**. LPS-stimulated macrophages were additionally treated with 5 μM nigericin for 45 min to measure IL-1β level in the cell culture supernatant **(D)**. The concentration of LH2171 or other lactic acid bacteria was 10 μg/mL unless otherwise indicated. Data are shown as mean + SD (*n* = 3) and analyzed by Tukey-Kramer's (^a−c^*P* < 0.05) **(A)** or Dunnett's test (*P < 0.05, **P < 0.01) **(B–D)**, which compared the LPS-treated group **(C)** or LPS + nigericin-treated group **(B,D)** with the other groups.

To investigate the anti-inflammatory properties of LH2171 in peritoneal macrophages, we next examined inflammatory cytokine secretion. LPS stimulation induced the secretion of IL-6 into the medium (Figure 1A) but did not induce the secretion of IL-1β (Figure 1B). Short-term treatment with nigericin, a proton ionophore that promotes IL-1β maturation, induced IL-1β secretion in LPS-stimulated peritoneal macrophages (Figure 1B). LH2171 treatment slightly induced IL-6 production in peritoneal macrophages (Figure 1A). However, secretion of both IL-6 and IL-1β cytokines from LPS-stimulated macrophages was significantly suppressed by LH2171 pretreatment (10 μg/mL or less) (Figures 1A,B). A similar result was observed for TNF-α secretion (Supplementary Figure 3). These results suggested that LH2171 exerted anti-inflammatory properties in LPS-stimulated macrophages through mechanisms other than those involved in anti-proliferative or cytotoxic effects.

To evaluate the specificity of the suppressive effect of LH2171 on cytokine production, we investigated whether different strains of several *Lactobacillus* species would affect IL-6 and IL-1β secretion of peritoneal macrophages. Although LPA1149^T^ did not affect IL-6 secretion, the other strains we assessed (LH1120^T^, LPL8131^T^, LAC1132^T^) significantly suppressed LPS-induced IL-6 secretion (Figure 1C). However, IL-1β secretion was suppressed by LH1120^T^, LPA1149^T^, and LPL8131^T^, but not by LAC1132^T^ (Figure 1D). Among these strains, LH1120^T^, a type strain of *L. helveticus*, had the most prominent inhibitory effect (Figures 1C,D). The suppressive effect of LH2171 on IL-6 and IL-1β secretion was similar to that of LH1120^T^ (Figures 1C,D). Therefore, the anti-inflammatory effect of *L. helveticus* strains would be relatively stronger than that of other bacterial strains.

In our experiments, some strains of lactobacilli exerted different effects on IL-6 and IL-1β secretion. Although LPA1149^T^ did not suppress IL-6 secretion, it inhibited IL-1β secretion (Figures 1C,D). LAC1132^T^ significantly suppressed IL-6 secretion, but it did not affect IL-1β secretion (Figures 1C,D). In our experimental conditions, IL-1β maturation and secretion were caused by nigericin, which is an NLRP3 inflammasome activator. Furthermore, these results implied that some lactobacilli might prevent (or aggravate) inflammasome activation. To evaluate the effect of LH2171 on inflammasome activation, we assessed whether the addition of LH2171 immediately before nigericin treatment could suppress IL-1β secretion from LPS-pretreated peritoneal macrophages. However, LH2171 treatment of LPS-pretreated macrophages did not suppress nigericin-stimulated IL-1β secretion (Supplementary Figure 4), indicating that LH2171 would not inhibit inflammasome activation.

### LH2171 Inhibits Pro-inflammatory Gene Expression in LPS-Stimulated Peritoneal Macrophages

**Figure 2:**
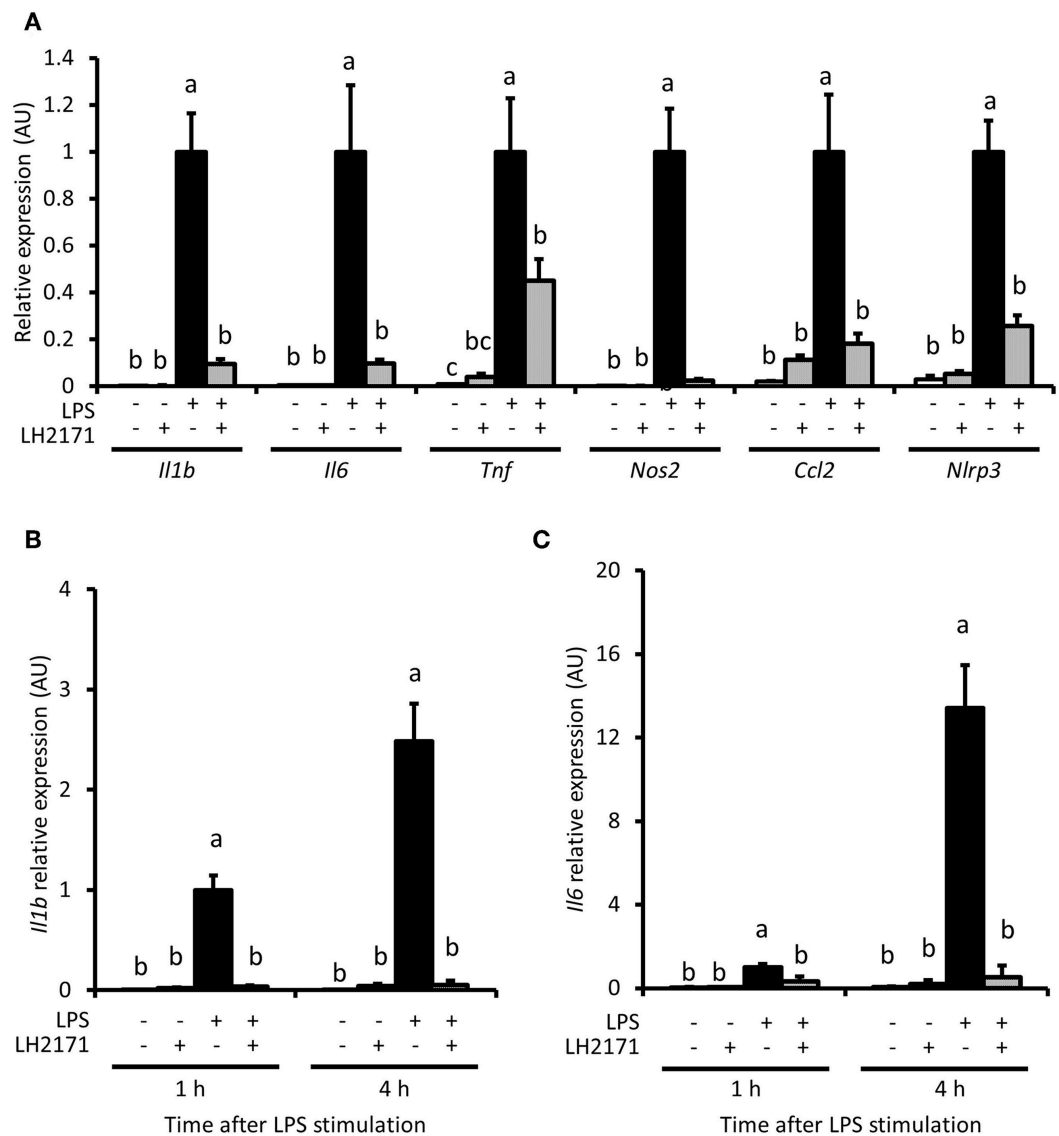
LH2171 inhibits pro-inflammatory gene expression in LPS-stimulated peritoneal macrophages. The peritoneal macrophages were either pre-incubated with or without 10 μg/mL LH2171 for 4 h and stimulated with 1 μg/mL LPS for 16 h, after which *Il1b, Il6, Tnf*, *Nos2, Ccl2*, and *Nlrp3* mRNAs were quantified by qPCR assay **(A)**. Expression of *Il1b* mRNA **(B)** and *Il6* mRNA **(C)** were analyzed after 1 and 4 h after LPS addition. The mRNA expression was determined relative to the mean value of LPS-treated control at 16 h **(A)** or at 1 h **(B,C)**. Data are shown as mean + SD (*n* = 3) and analyzed by Tukey-Kramer's (^a−c^*P* < 0.05), which compared in the same time point.

We evaluated whether LH2171 could inhibit expression of pro-inflammatory genes, including *Il1b* and *Il6*, in peritoneal macrophages. *Il1b, Il6* and other pro-inflammatory genes, such as *Tnf*, *Nos2, Ccl2*, and *Nlrp3*, were up-regulated by a 16-h LPS treatment (Figure 2A). Further, LH2171 pre-treatment significantly inhibited the increased expression of these genes in LPS-stimulated macrophages (Figure 2A). A significant suppressive effect of LH2171 pre-treatment on *Il1b* and *Il6* gene expression could be observed as early as 1 h and at 4 h after LPS stimulation (Figures 2B,C).

### LH2171 Inhibits NF-κB/MAPK Signaling Activation in LPS-Stimulated Peritoneal Macrophages

**Figure 3:**
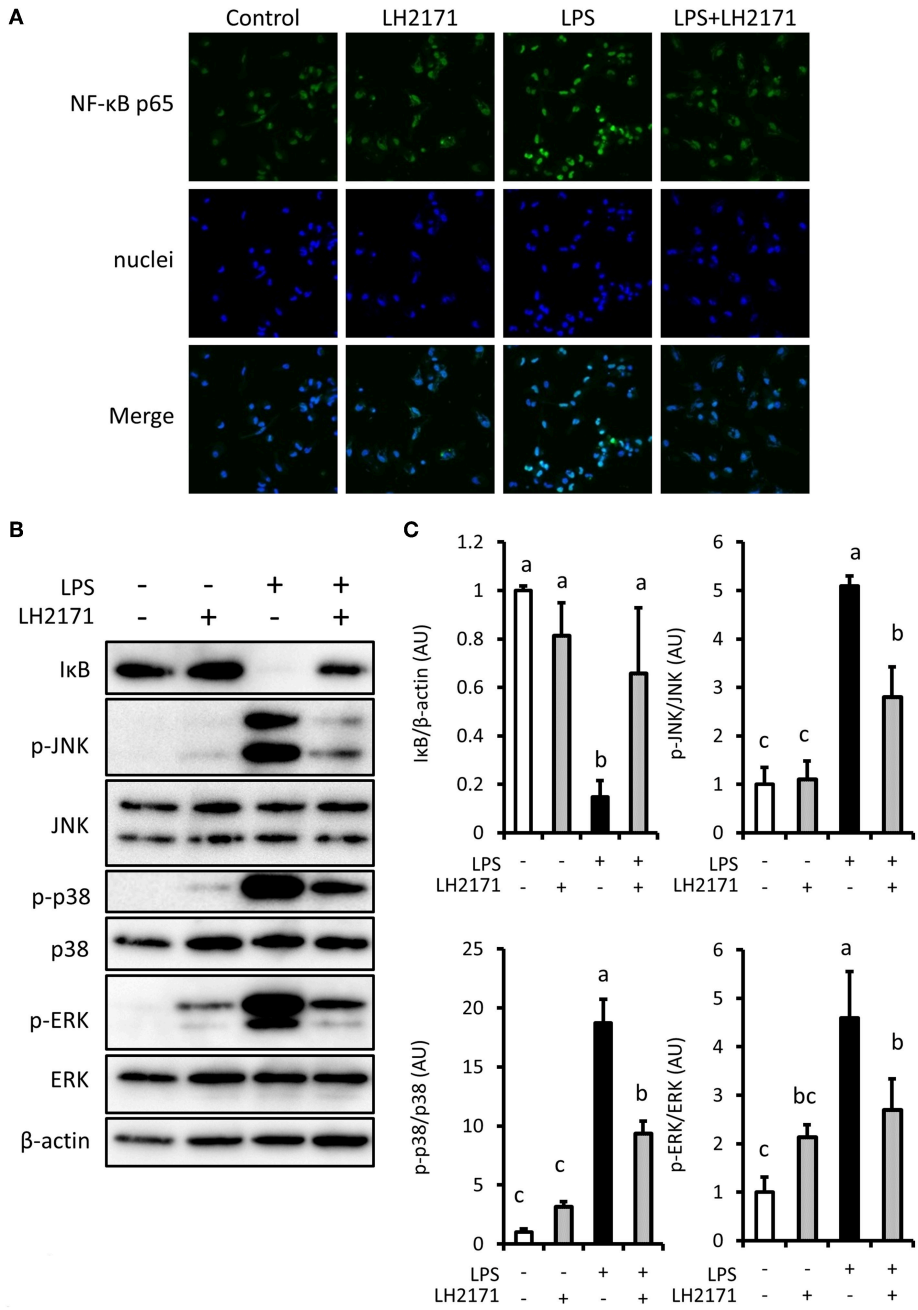
LH2171 inhibited the activation of NF-κB/MAPKs signaling in LPS-stimulated peritoneal macrophages. The peritoneal macrophages were pre-incubated with or without 10 μg/mL LH2171 for 4 h and stimulated with 1 μg/mL LPS for 15 min, after which NF-κB p65 subunit (Green) and nuclei (Blue) were visualized using a confocal fluorescence microscope **(A)** and IκB, JNK, p-JNK, p38, p-p38, ERK, p-ERK, and β-tubulin were detected by western blot analysis **(B)**. Densitometric quantification of IκB/β-tubulin ratio, p-JNK/JNK ratio, p-p38/p38 ratio, and p-ERK/ERK ratio was shown **(C)**. The quantified values were determined relative to the mean values of the untreated control. Data are shown as mean + SD (*n* = 3) and analyzed by Tukey-Kramer's (^a−c^*P* < 0.05).

Pro-inflammatory gene expression in LPS-stimulated macrophages is induced by activation of NF-κB and MAPK mediated signaling pathways (22). Therefore, we investigated the effect of LH2171 on NF-κB and MAPK mediated signaling pathway activation. LPS treatment changed the intracellular localization of the NF-κB p65 subunit, which contains a transcriptional activation domain (Figure 3A). It also caused degradation of IκB (Figures 3B,C), which is an inhibitor of NF-κB. These changes were inhibited by LH2171 pre-treatment (Figures 3A–C). LH2171 also inhibited phosphorylation of major MAPK signaling molecules (JNK, p38, ERK) (Figures 3B,C). These results suggested that LH2171 could inhibit activation of LPS-stimulated NF-κB/MAPKs signaling in peritoneal macrophages.

### LH2171 Induces Expression of Negative Regulators of NF-κB/MAPKs Signaling in Peritoneal Macrophages

**Figure 4:**
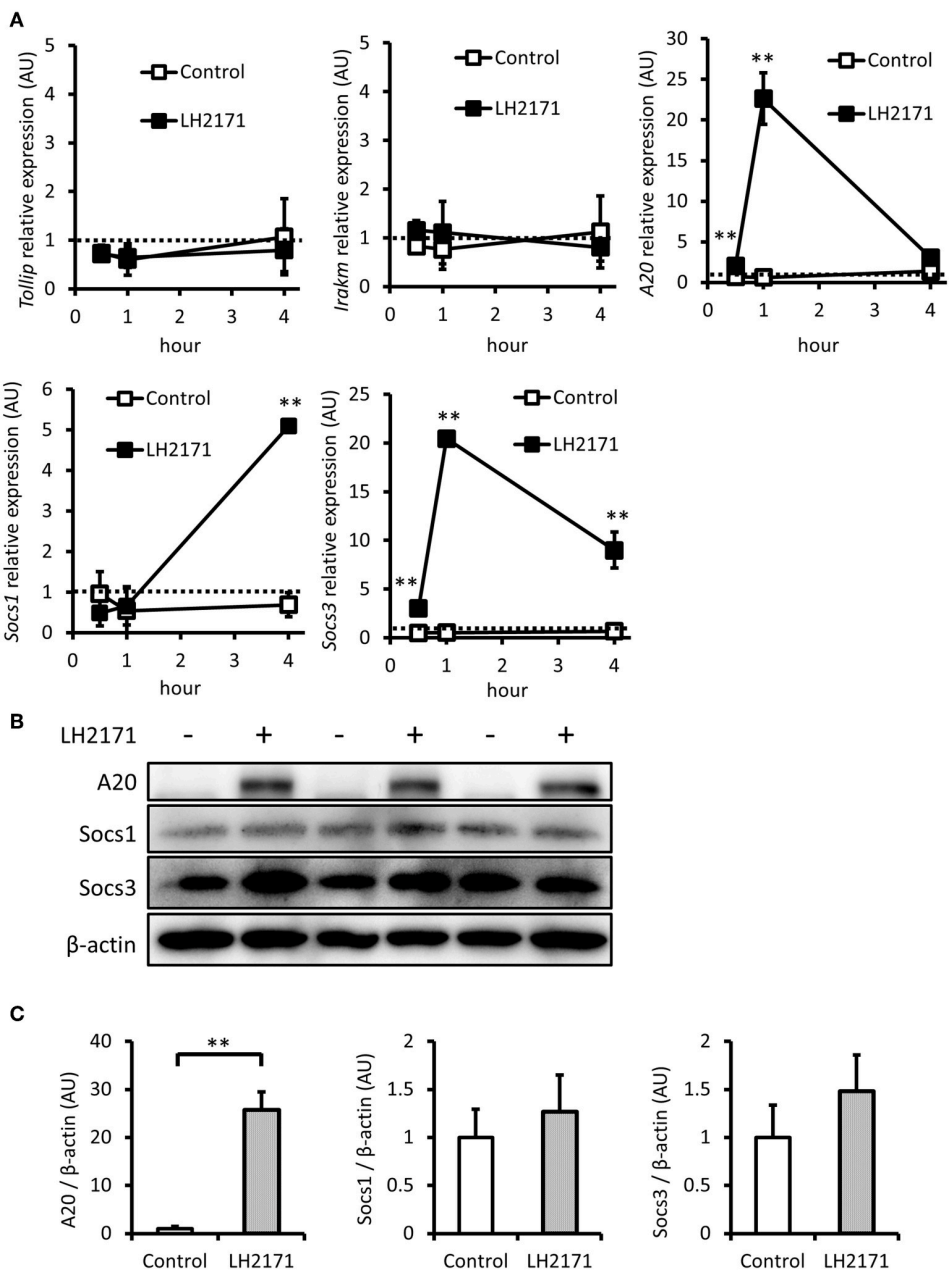
LH2171 induced the expression of negative regulators of NF-κB/MAPKs signaling in peritoneal macrophages. The peritoneal macrophages were pre-incubated with or without 10 μg/mL LH2171 for 30 min, 1 and 4 h, after which *Tollip, Irakm, A20, Socs1*, and *Socs3* mRNAs were quantified by qPCR assay **(A)**. The peritoneal macrophages were pre-incubated with or without 10 μg/mL LH2171 for 4 h, after which A20, Socs1, Socs3, and β-tubulin were detected by western blot analysis **(B)**. Densitometric quantification of A20/β-tubulin ratio, Socs1/β-tubulin ratio, and Socs3/β-tubulin ratio was shown **(C)**. The quantified values were determined relative to the mean values of untreated control at 0 h **(A)** or at 4 h **(C)**. Data are shown as mean + (±) SD (*n* = 3) and analyzed by Student's *t*-test (**P < 0.01), which compared the LH2171-treated group with untreated control at the same time point.

The activation of both NF-κB and MAPK mediated signaling pathways by LPS can be suppressed by various negative regulators, including Tollip, IRAK-M, A20, SOCS1, and SOCS3 (23). Therefore, we investigated whether LH2171 pretreatment would affect gene expression of these negative regulators before LPS stimulation. LH2171 treatment for 4 h had little effect on the expression of *Tollip* and *Irakm* (Figure 4A). However, *A20* and *Socs3* expression levels were increased 1 h after the addition of LH2171, and *Socs1* increased 4 h after treatment (Figure 4A). Subsequently, we evaluated protein expression levels of A20, Socs1, and Socs3. After a 4-h treatment of LH2171, the protein level of A20 was significantly increased; however, Socs1 and Socs3 were not changed (Figures 4B,C). These results suggested that A20 may contribute to the anti-inflammatory effect of LH2171 in peritoneal macrophages.

### LH2171 Cell Wall Exerts Anti-inflammatory Effect in Peritoneal Macrophages

**Figure 5:**
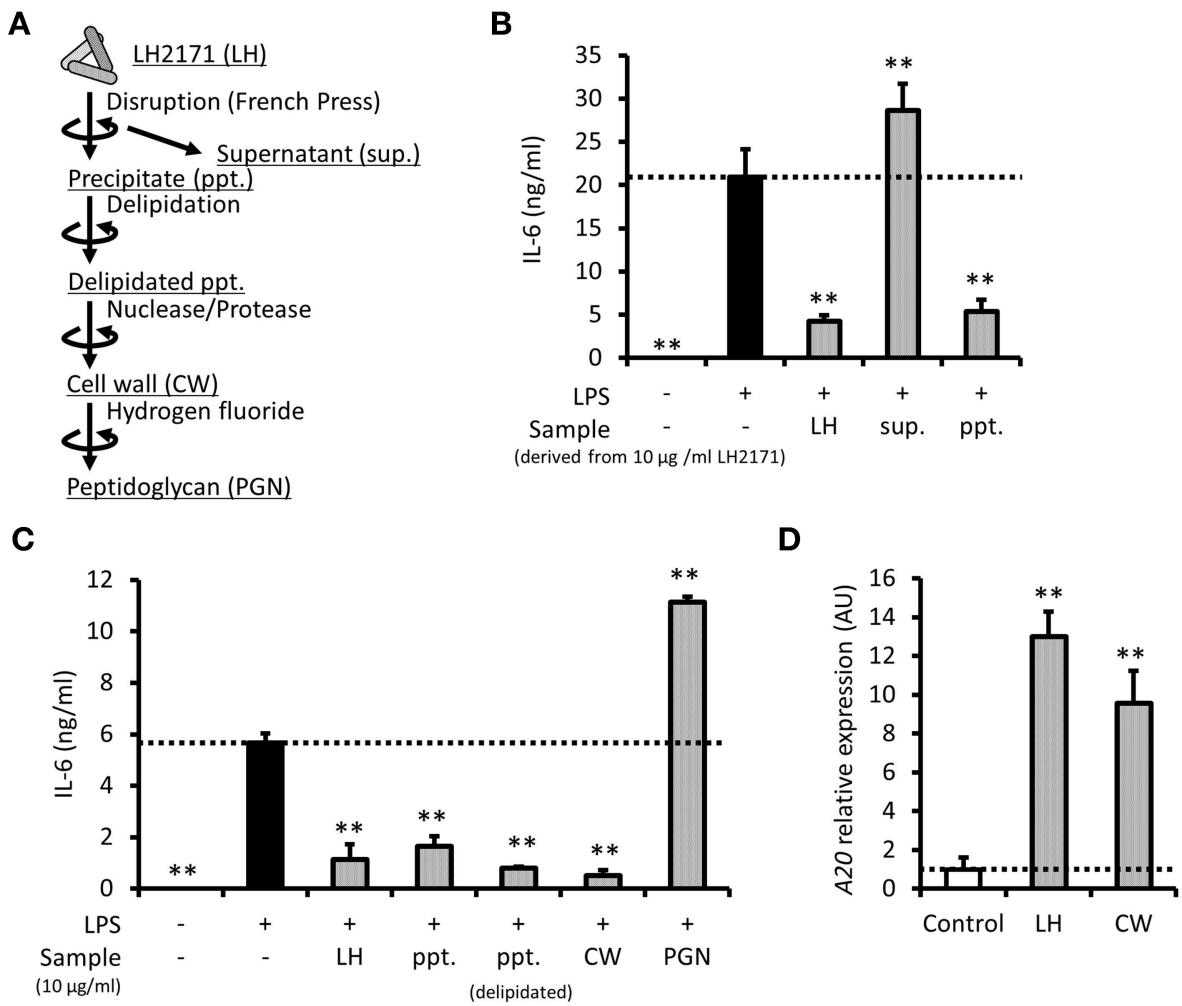
LH2171 cell wall exerted an anti-inflammatory effect on peritoneal macrophages. The preparation steps for cell components of LH2171 were shown **(A)**. The peritoneal macrophages were pre-incubated with supernatant (sup.) or precipitate (ppt.), which were derived from 10 μg/mL LH2171 disrupted by French pressure cell, for 4 h and stimulated with 1 μg/mL LPS for 16 h, after which IL-6 level in the culture supernatant was determined **(B)**. The peritoneal macrophages were pre-incubated with 10 μg/mL cell components of LH2171 [ppt., delipidated ppt., cell wall (CW) or peptidoglycan (PGN)] for 4 h and stimulated with 1 μg/mL LPS for 16 h, after which IL-6 level in the culture supernatant was determined **(C)**. The peritoneal macrophages were incubated with LH2171 or CW for 1 h, after which *A20* mRNA was quantified by qPCR assay **(D)**. The *A20* mRNA expression was determined relative to the mean value of untreated control. Data are shown as mean + (±) SD (*n* = 3) and analyzed by Dunnett's test (**P < 0.01), which compared the LPS-treated group **(B,C)** or untreated group **(D)** with the other groups.

To clarify which component of LH2171 contributes to its anti-inflammatory role, the cell components of LH2171 (Figure 5A) were fractionated and evaluated for their suppressive effects on cytokine production. First, the water-soluble (supernatant) and water-insoluble (precipitate) fractions were separated from mechanically disrupted LH2171 cells and examined. Further, IL-6 secretion in LPS-stimulated peritoneal macrophages was suppressed by precipitates, similar to whole LH2171 cells, but not by supernatants (Figure 5B), indicating that anti-inflammatory LH2171 components were present in the water-insoluble fraction. One of the major water-insoluble bacterial components is the cell wall. Therefore, we purified the LH2171 cell wall from the precipitate and evaluated its anti-inflammatory properties. Both the purified cell wall and delipidated precipitate, which is an intermediate in the process of cell wall purification, effectively suppressed IL-6 secretion, similar to LH2171 cells (Figure 5C). In contrast, the peptidoglycan, which is a major structural component of the bacterial cell wall, caused an increase in IL-6 secretion from LPS-stimulated peritoneal macrophages (Figure 5C). These results indicated that some cell wall components in LH2171, other than the peptidoglycan, have anti-inflammatory effects on peritoneal macrophages. Furthermore, we demonstrated that the cell wall fraction of LH2171 induced *A20* expression as effectively as LH2171 (Figure 5D), suggesting that A20 might mediate the anti-inflammatory effect of this fraction.

### LH2171 Regulates *A20* Gene Expression via TLR2 Signaling

**Figure 6:**
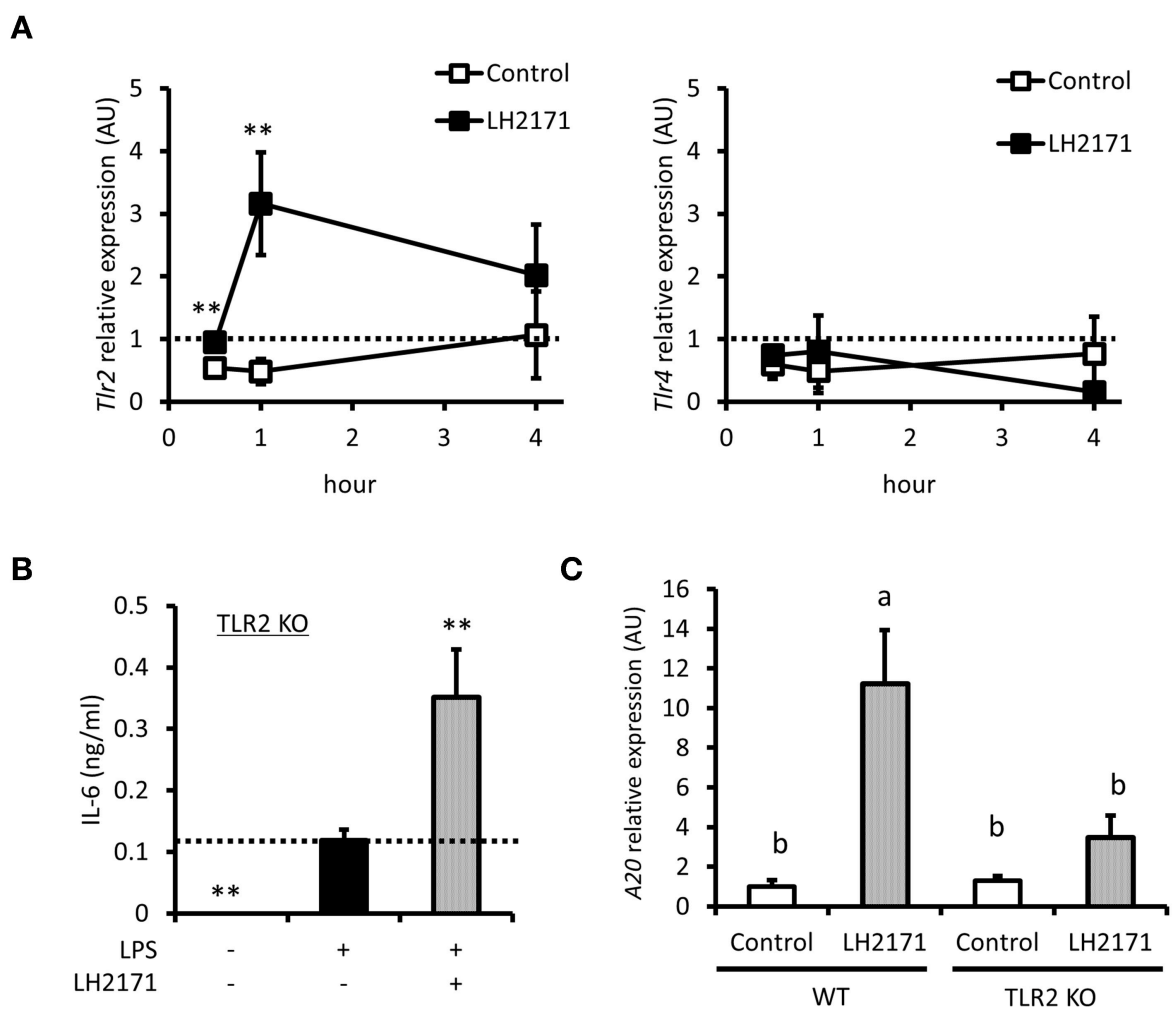
LH2171 regulated *A20* gene expression via TLR2 signal. The peritoneal macrophages were incubated with or without 10 μg/mL LH2171 for 30 min, 1 and 4 h, after which *Tlr2* and *Tlr4* mRNAs were quantified by qPCR assay **(A)**. The peritoneal macrophages derived from TLR2 knock-out (KO) mice were pre-incubated with or without LH2171 for 4 h and stimulated with 1 μg/mL LPS for 16 h, after which IL-6 level in the culture supernatant was determined **(B)**. The peritoneal macrophages derived from wild-type (WT) or TLR2 KO mice were incubated with or without LH2171 for 1 h, after which *A20* mRNA was quantified by qPCR assay **(C)**. Data are shown as mean + (±) SD (*n* = 3). The data of *Tlr2* and *Tlr4* expression were determined relative to the untreated control at 0 h and analyzed by student's *t*-test (**P < 0.01), which compared LH2171-treated group with untreated control in the same time point **(A)**. The data of IL-6 level in the culture supernatant were analyzed by Dunnett's test (**P < 0.01), which compared LPS-treated group with the other groups **(B)**. The data of *A20* mRNA expression were determined relative to the untreated control of WT and analyzed by Tukey-Kramer's (^a,b^*P* < 0.05) **(C)**.

TLR2 is known to recognize cell wall components of Gram-positive bacteria, which is a pattern recognition receptor expressed on the cell surface (24). Some Gram-positive bacteria were reported to exert anti-inflammatory effects through the TLR2 signaling pathway (25, 26). These reports and the fact that LH2171 is a Gram-positive bacterium prompted us to investigate whether TLR2 signaling regulated the anti-inflammatory effect of LH2171. TLR2 gene expression was increased by LH2171 treatment; however, expression of TLR4, another cell surface pattern recognition receptor, was not affected (Figure 6A). We then used TLR2 KO mice to evaluate the effect of LH2171 on cytokine production in TLR2-depleted peritoneal macrophages. LH2171 did not suppress IL-6 secretion in peritoneal macrophages of TLR2 KO mice (Figure 6B). Moreover, the increase in *A20* mRNA expression induced by LH2171 treatment was significantly weaker in peritoneal macrophages of TLR2 KO mice than wild-type mice (Figure 6C). These results indicated that LH2171 induction of *A20* expression and suppression of IL-6 secretion in peritoneal macrophages are mediated by TLR2 signaling.

## Discussion

Earlier studies have reported that intraperitoneal injection of LH2171 improved clinical symptoms and decreased serum IL-6 level in mice with collagen-induced arthritis (CIA) (18, 19). LH2171 injection also inhibited development of experimental autoimmune encephalomyelitis (EAE) and IL-6 expression in inguinal lymph nodes in mice immunized with a peptide containing amino acid sequence (139–151 residues) of the proteolipid protein (20). In addition, LH2171 prevented IL-6 secretion in the macrophage cell line RAW264.7 and dendritic cell line DC2.4 (20). These observations prompted us to investigate the precise mechanism of LH2171 anti-inflammatory activity. We used peritoneal antigen-presenting cells, which would be influenced by an intraperitoneal injection of LH2171, as a model. In this study, we demonstrated that LH2171 inhibited the secretion of IL-6 and IL-1β, which promotes the pathogenesis of autoimmune diseases as well as IL-6 (27, 28), in peritoneal macrophages. LH2171 also suppressed the expression of pro-inflammatory genes, including *Il1b* and *Il6*, in LPS-stimulated peritoneal macrophages. Moreover, LH2171 inhibited activation of the NF-κB/MAPKs signaling pathways in these cells. These results suggested that LH2171 has the potential to improve the inflammatory status of primary macrophages in mouse models of inflammatory diseases.

Some lactobacilli were reported to modulate macrophage inflammatory responses (29). Some of these reports focused on IL-10 production in macrophages as a lactobacilli anti-inflammatory mechanism. For example, *L. rhamnosus* GG, which is widely used as a probiotic, was reported to increase IL-10 production and, conversely, decrease TNF-α production in an LPS-stimulated macrophage cell line (30). *L. plantarum* OLL2712, a bacterial strain selected to strongly induce IL-10 secretion in macrophages, demonstrated anti-inflammatory effects in type 2 diabetic mice (31). Furthermore, *L. helveticus* NS8, which belongs to the same species as LH2171, exerted anti-inflammatory effects by inducing IL-10 secretion in macrophages (32). Therefore, we assessed whether LH2171 would affect IL-10 secretion in peritoneal macrophages. However, IL-10 gene expression and secretion were decreased by LH2171 treatment in LPS-stimulated peritoneal macrophages (Supplementary Figure 5). Moreover, anti-IL-10 blocking antibody did not diminish the suppressive effect of LH2171 on IL-6 production (Supplementary Figure 6). These observations suggested that some factors other than IL-10 might be important for LH2171 to exert its anti-inflammatory effects in macrophages.

The TLR4-MyD88-IRAK1/4-TRAF6 signaling pathway, which is important for the LPS-induced NF-κB/MAPKs activation, is known to be controlled by various negative regulators. For instance, SOCS1 is one of these regulators. SOCS1 has been reported to induce degradation of TIRAP, an adaptor protein involved in bridging MyD88 to TLR4 and disturbing formation of the TLR4/MyD88 complex (33). Tollip interacts with IRAK1 and inhibits its self-phosphorylation and kinase activity required for the subsequent TRAF6 activation (34). IRAK-M blocks the dissociation of IRAK1/4 from MyD88 and inhibits its downstream signaling (35). A20 and SOCS3 promote TRAF6 degradation and prevent subsequent NF-κB/MAPKs activation (33, 36). Some lactobacilli and bifidobacteria have been reported to induce the gene expression of these negative regulators and inhibit the inflammatory signal (25, 26, 37–39). In the present study, we demonstrated that the mRNA expression of *Tollip* and *Irakm* did not change, but that of *A20, Socs1*, and *Socs3* was increased in peritoneal macrophages by LH2171 treatment. A corresponding increase was also observed in A20 protein levels at 4 h after LH2171 treatment, but not in Socs1 and Socs3 protein levels. We also assessed A20, Socs1, and Socs3 protein levels at a later time point (20 h after LH2171 treatment). Socs3 protein level was increased about 2 times by LH2171 treatment after 20 h (Supplementary Figure 7), suggesting that Socs3 could contribute to the anti-inflammatory effect of LH2171. However, A20 protein level was also increased about 4 times by LH2171 treatment after 20 h (Supplementary Figure 7). Although Socs3 was increased by LH2171 treatment, A20 was increased faster and more substantially than Socs3. Therefore, we consider that A20 would be more important than Socs3 for the anti-inflammatory properties of LH2171 in peritoneal macrophages.

Some Gram-positive bacteria were reported to induce A20 mRNA expression via TLR2 signaling. It was demonstrated that *Bifidobacterium* (*B*.) *longum* BB536 and *B. breve* M16-V up-regulated the mRNA expression of A20 in porcine intestinal epithelial cells, and this up-regulation was partly reversed by an anti-TLR2 blocking antibody (26). It was also shown that *L. paracasei* induced A20 gene expression in the human monocytic cell line THP-1, which was partially reduced by TLR2 blocking (25) as well as *B. longum* BB536 and *B. breve* M16-V. *L. paracasei*-induced A20 expression was mimicked by Pam3CSK4, which is a TLR2 agonist (25). To examine whether TLR2 signaling is critical for the LH2171-mediated increase in the A20 expression level, we used peritoneal macrophages from TLR2 KO mice and investigated the effect of LH2171 on *A20* gene expression. The *A20* expression level in LH2171-treated TLR2 KO macrophages was significantly lower than that in LH2171-treated wild-type macrophages. These results suggest that TLR2 signaling contributes to the induction of *A20* gene expression in LH2171-treated peritoneal macrophages. Several *L. helveticus* strains are reported to have anti-inflammatory properties, such as *L. helveticus* NS8 mentioned above (32). We have, for the first time, demonstrated that one of these strains, LH2171, could regulate A20 expression via TLR2 signaling.

One of the most common activators of TLR2 signaling in Gram-positive bacterial components is lipoteichoic acid (LTA). LTA is a glycopolymer connected to the cell membrane of Gram-positive bacteria and acts as a TLR2 ligand on various innate immune cells (40). LTA isolated from a *Lactobacillus* strain was reported to demonstrate anti-inflammatory effects in LPS-stimulated THP-1 cells through a TLR2-dependent pathway (41). However, in the present study, the delipidated precipitate and cell wall fraction derived from LH2171, which do not contain cell membrane fractions that include LTA (16), showed an anti-inflammatory effect in peritoneal macrophages. These fractions were found to be similarly effective as the intact LH2171 cell. This result indicates that the anti-inflammatory components of LH2171 are different from LTA.

Our results suggested that the cell wall fraction of LH2171 included the anti-inflammatory factor(s) for macrophages. The cell wall of Gram-positive bacteria is mainly composed of peptidoglycan. Various reports have suggested peptidoglycan as one of the TLR2 ligands (42). Moreover, muropeptide, which is a peptidoglycan fragment, was reported to exert anti-inflammatory effects through nucleotide-binding oligomerization domain 2 (NOD2) signaling (43). However, in the present study, the peptidoglycan derived from LH2171 aggravated the inflammatory status in the LPS-stimulated peritoneal macrophages. In addition, under our experimental conditions, the NOD2 ligand, muramyl dipeptide, exacerbated the inflammatory response by increasing the macrophage IL-6 production level (Supplementary Figure 8). Therefore, peptidoglycan and its fragment did not contribute to the anti-inflammatory effects of LH2171 in macrophages.

Peptidoglycan-attached glycopolymers, such as wall teichoic acid or other cell wall polysaccharide, also constitute the cell wall of Gram-positive bacteria (40). Removal of peptidoglycan-linked glycopolymers from the cell wall of LH2171 by hydrofluoric acid diminished the anti-inflammatory potential, indicating that the wall teichoic acid or cell wall polysaccharide might act as anti-inflammatory factors. The wall teichoic acid has been shown to contribute to TLR2 signaling activation in some lactobacilli treatment (16), which could also possibly up-regulate A20 expression in macrophages. To elucidate whether peptidoglycan-attached glycopolymers in LH2171 have anti-inflammatory effects on peritoneal macrophages, we used a peptidoglycan-lytic enzyme, mutanolysin. It was used to remove peptidoglycan and obtain intact peptidoglycan-attached glycopolymers from LH2171. However, mutanolysin-treatment diminished the inhibitory effect of LH2171 and its cell wall on IL-6 secretion in peritoneal macrophages (Supplementary Figure 9). As described above, peptidoglycan fragments might aggravate the inflammatory response of LPS-stimulated peritoneal macrophages, so that the contamination of peptidoglycan fragments produced by mutanolysin-treatment would abrogate the anti-inflammatory effects. Therefore, it would be important to further examine whether the purified peptidoglycan-attached glycopolymers of LH2171 would exert anti-inflammatory effects in peritoneal macrophages.

In the present study, we have shown that LH2171 inhibited inflammation caused by LPS treatment using mouse peritoneal macrophages. These results suggested that LH2171 might be effective for the treatment of sepsis. However, in the present study, all results were obtained from *in vitro* experiments and, therefore, we could not evaluate the precise effect of LH2171 *in vivo*. To evaluate whether the anti-inflammatory mechanisms observed in this study could be extrapolated *in vivo, in vivo* experiments, such as a sepsis model, should be conducted in the future.

In the present study, we demonstrated that LH2171 inhibited activation of the NF-κB/MAPKs signaling pathway and secretion of pro-inflammatory cytokines, such as IL-1β and IL-6, in LPS-stimulated peritoneal macrophages. We also showed that LH2171 and its cell wall fraction induced A20 expression, a negative regulator of NF-κB/MAPKs signaling, in macrophages. Moreover, LH2171-induced up-regulation of A20 was mediated by TLR2 signal. Further, pro-inflammatory cytokines produced by antigen-presenting cells influence inflammatory disease progression. Thus, the anti-inflammatory mechanisms observed in peritoneal macrophages treated with LH2171 might contribute to the beneficial effects of LH2171 on these inflammatory diseases.

## Ethics Statement

This study was carried out in accordance with the recommendations of the guidelines of the Bioscience Committee of Hokkaido University (Sapporo, Hokkaido, Japan). The protocol was approved by the Animal Care and Use Committee of Hokkaido University.

## Author Contributions

MK carried out experimental work. MK analyzed data and wrote the manuscript. MM and TM supervised the study and reviewed the manuscript. All authors designed the study. All authors read and approved the final manuscript.

### Conflict of Interest Statement

MK and MM are employed by Megmilk Snow Brand Co., Ltd. The content of this paper was neither influenced nor constrained by the fact. The remaining author declares that the research was conducted in the absence of any commercial or financial relationships that could be construed as a potential conflict of interest.

## Abbreviations

LH2171: *Lactobacillus helveticus* SBT2171
LPS: lipopolysaccharide
TLR: toll-like receptor
NF-κB: nuclear factor-kappa B
MAPK: mitogen-activated protein kinase
CIA: collagen-induced arthritis
EAE: experimental autoimmune encephalomyelitis
IL: interleukin
PAMPs: pathogen-associated molecular patterns
DAMPs: damage-associated molecular patterns
LAB: lactic acid bacteria
LTA: lipoteichoic acid
CW: cell wall
PGN: peptidoglycan
NOD2: nucleotide-binding oligomerization domain 2.
