# Supplementary material for: Lactobacillus helveticus SBT2171 Induces A20 Expression via Toll-Like Receptor 2 Signaling and Inhibits the Lipopolysaccharide-Induced Activation of Nuclear Factor-kappa B and Mitogen-Activated Protein Kinases in Peritoneal Macrophages
Source: Front Immunol. 2019 Apr 17;10:845. doi: 10.3389/fimmu.2019.00845 (PMC6478895; doi:10.3389/fimmu.2019.00845)
Supplement: Supplementary file 1 [file Data_Sheet_1.docx]

Supplementary Material

*Lactobacillus helveticus* SBT2171 Induces A20 Expression *via* Toll-like Receptor 2 Signaling and Inhibits the Lipopolysaccharide-induced Activation of Nuclear Factor-kappa B and Mitogen-activated Protein Kinases in Peritoneal Macrophages

Michio Kawano, Masaya Miyoshi, Tadaaki Miyazaki^*^

*** Correspondence:** Tadaaki Miyazaki: [miyazaki@pop.med.hokudai.ac.jp](mailto:miyazaki@pop.med.hokudai.ac.jp)

**Supplementary Figure**

**
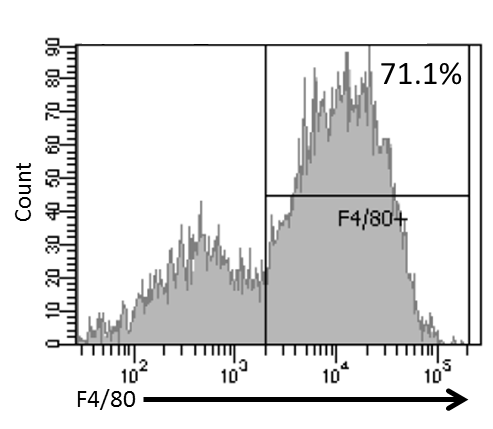
**

**Supplementary Figure 1**

**The ratio of F4/80 positive cells per the peritoneal exudate adherent cells**

The peritoneal exudate cells were seeded on plate and incubated for three hours. The adherent cells were washed once with PBS and detached with trypsin/EDTA. The adherent cells were treated with Zombie Aqua (Biolegend, San Diego, CA, USA) live–dead discriminating dye. These cells were treated with anti-mouse CD16/32 antibody (Biolegend) and then stained with Brilliant Violet 421^TM^-linked anti-F4/80 antibody (Biolegend) or corresponding isotype control (Biolegend). The stained cells were fixed with paraformaldehyde and analyzed with a flow cytometer (FACS Canto II, BD bioscience, Franklin Lakes, NJ, USA). The ratio of F4/80 positive cells per the live adherent cells was calculated.

**
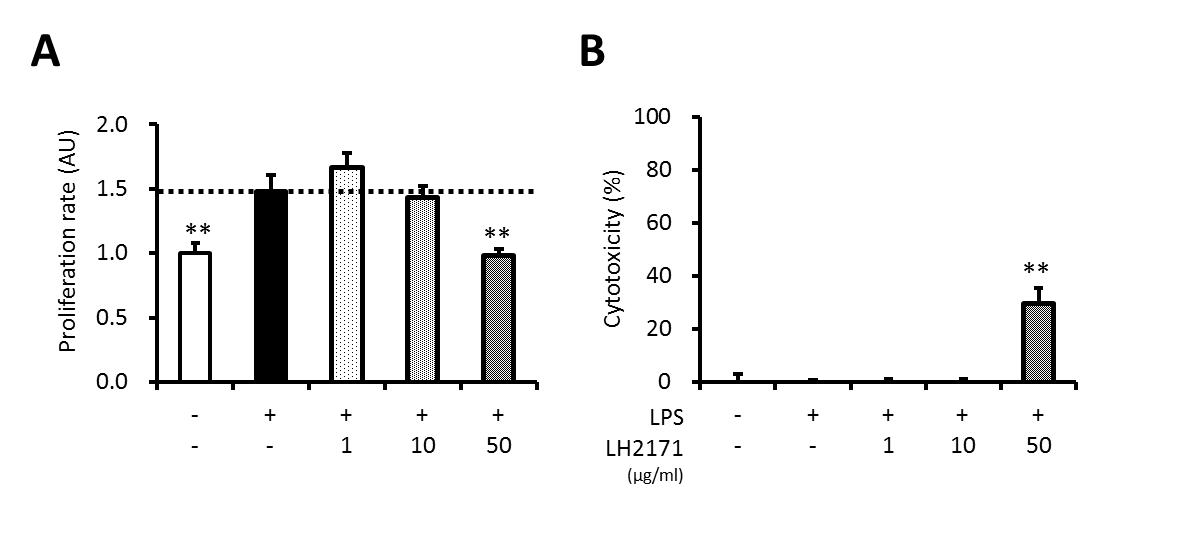
**

**Supplementary Figure 2**

**LH2171 does not affect cell proliferation or cell death in LPS-stimulated peritoneal macrophages at a concentration of 10 µg/ml or less.**

The peritoneal macrophages were pre-incubated with or without LH2171 for 4 hours and stimulated with 1 µg/ml LPS for 16 hours, after which cell proliferation rate **(A)** and cytotoxicity **(B)** were determined. The cell proliferation rate was determined relative to the mean value of untreated control **(A)**. The cytotoxicity was determined by calculating the percentage of LDH release relative to the maximum LDH release control **(B)**. Data are shown as mean + SD (n = 3) and analyzed by Dunnett’s test (***P* < 0.01), which compared the LPS-treated group with the other groups.


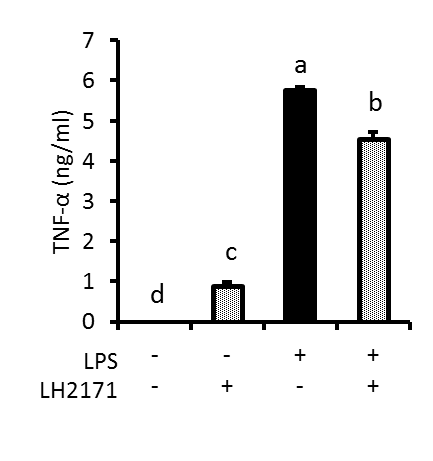


**Supplementary Figure 3**

**LH2171 inhibited secretion of TNF-α from LPS-stimulated peritoneal macrophages.**

The peritoneal macrophages were pre-incubated with or without 10 µg/ml LH2171 for 4 hours and stimulated with 1 µg/ml LPS for 16 hours, and subsequently TNF-α amount in the culture supernatant was measured by ELISA kit (Biolegend). Data are shown as mean + SD (n = 3) and analyzed by Tukey-Kramer’s (^a-d^*P* < 0.05).


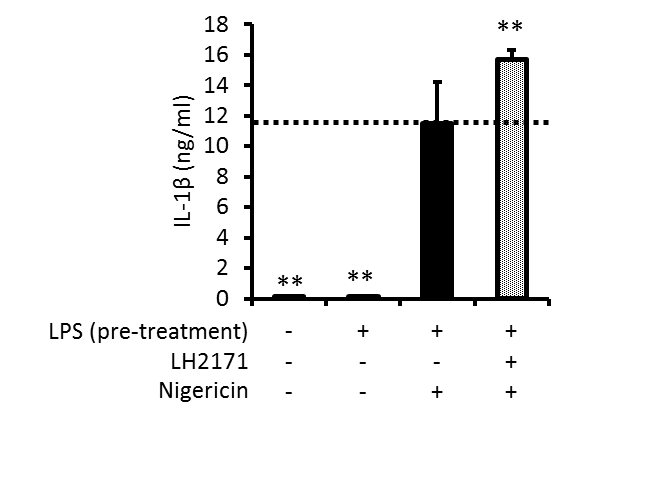


**Supplementary Figure 4**

**LH2171 did not inhibit IL-1β secretion from LPS-pretreated peritoneal macrophages.**

The peritoneal macrophages were pre-incubated with 1 µg/ml LPS for 16 hours and then treated with 10 µg/ml LH2171 for 2 hours. Subsequently these macrophages were additionally treated with 5 µM nigericin for 45 min to measure IL-1β amount in the cell culture supernatant. Data are shown as mean + SD (n = 3) and analyzed by Dunnett’s test (***P* < 0.01), which compared the LPS+nigericin-treated group with the other groups.


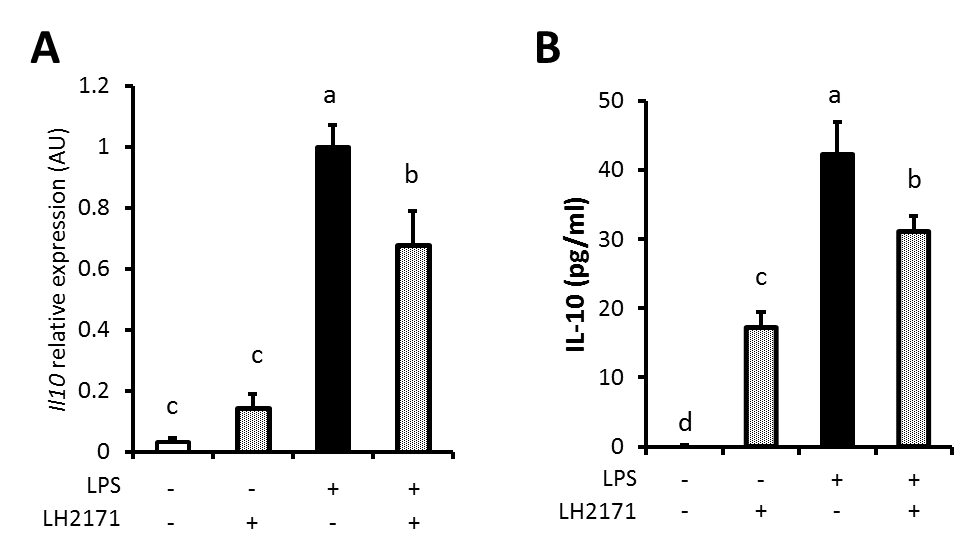


**Supplementary Figure 5**

**LH2171 inhibited *Il10* mRNA expression and IL-10 production in LPS-stimulated peritoneal macrophages.**

The peritoneal macrophages were pre-incubated with or without 10 µg/ml LH2171 for 4 hours and stimulated with 1 µg/ml LPS for 16 hours, after which *Il10* mRNA was quantified by a qPCR assay **(A)** and IL-10 amount in the cell culture supernatant was measured **(B)**. The *Il10* mRNA expression was determined relative to the mean value of LPS-treated control. Data are shown as mean + SD (n = 3) and analyzed by Tukey-Kramer’s (^a-d^*P* < 0.05).


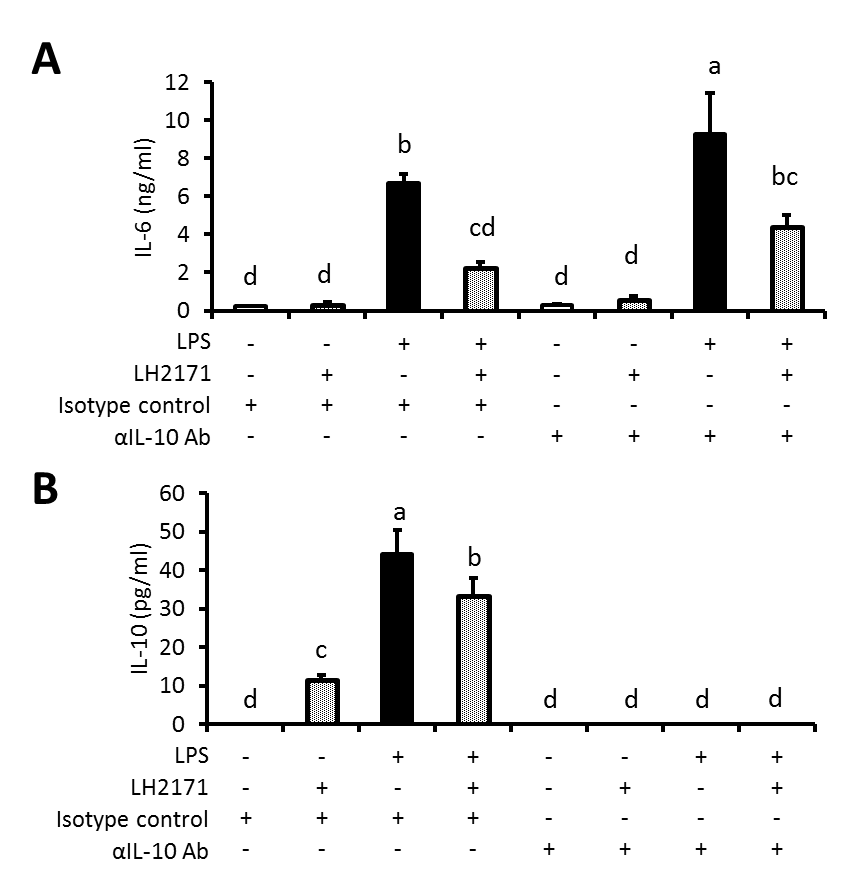


**Supplementary Figure 6**

**The treatment of IL-10 blocking antibody did not affect the inhibitory effect of LH2171 on IL-6 secretion from peritoneal macrophages.**

The peritoneal macrophages were pre-incubated with 10 µg/ml anti-IL-10 antibody (Tonbo Biosciences, San Diego, CA, USA) or corresponding isotype control (Biolegend) for 2 hours. These macrophages incubated with or without 10 µg/ml LH2171 for 4 hours and stimulated with 1 µg/ml LPS for 16 hours, after which IL-6 **(A)** or IL-10 **(B)** amount in the cell culture supernatant was measured by ELISA kits (**(A)** Biolegend, **(B)** R&D systems, Minneapolis, MN, USA). Data are shown as mean + SD (n = 3) and analyzed by Tukey-Kramer’s (^a-d^*P* < 0.05).


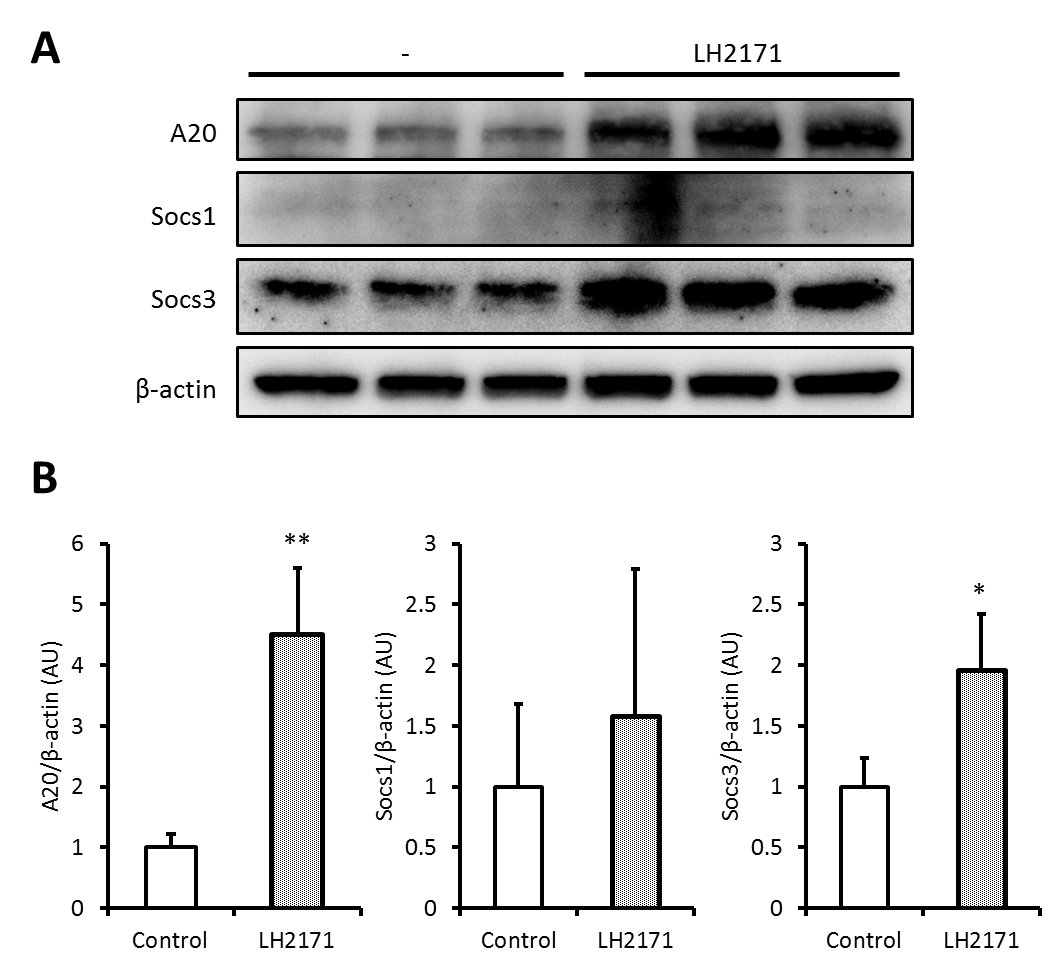


**Supplementary Figure 7**

**LH2171 treatment for 20 hours upregulated the protein level of A20 and Socs3 in peritoneal macrophages**

The peritoneal macrophages were incubated with or without 10 µg/ml LH2171 for 20 hours, after which the expression of A20, Socs1, Socs3, and β-tubulin was detected by western blot analysis **(A)**. The ratio of A20/β-tubulin, Socs1/β-tubulin, and Socs3/β-tubulin by the densitometric quantification was shown **(B)**. The quantified values were determined relative to the mean values of untreated control. Data are shown as mean + SD (n = 3) and analyzed by Student’s *t*-test (**P* < 0.05, ***P* < 0.01).

**
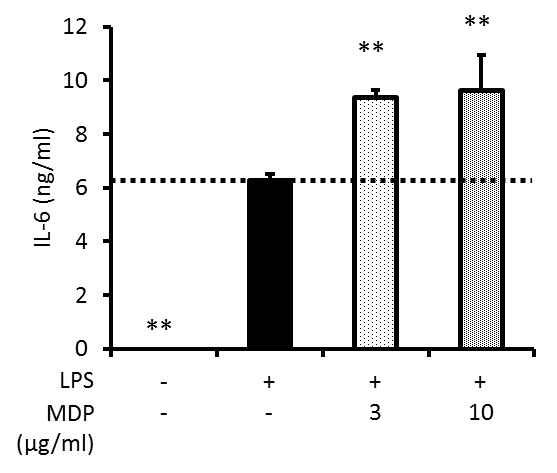
**

**Supplementary Figure 8**

**Muramyl dipeptide did not inhibit inflammatory cytokine production in LPS-stimulated peritoneal macrophages.**

The peritoneal macrophages were pre-incubated with 3 or 10 µg/ml muramyl dipeptide (MDP) for 4 hours and stimulated with 1 µg/ml LPS for 16 hours, after which IL-6 level in the culture supernatant was determined. MDP was purchased from Sigma Aldrich (St. Louis, MO, USA). Data are shown as mean + SD (n = 3) and analyzed by Dunnett’s test (***P* < 0.01), which compared the LPS-treated group with the other groups.


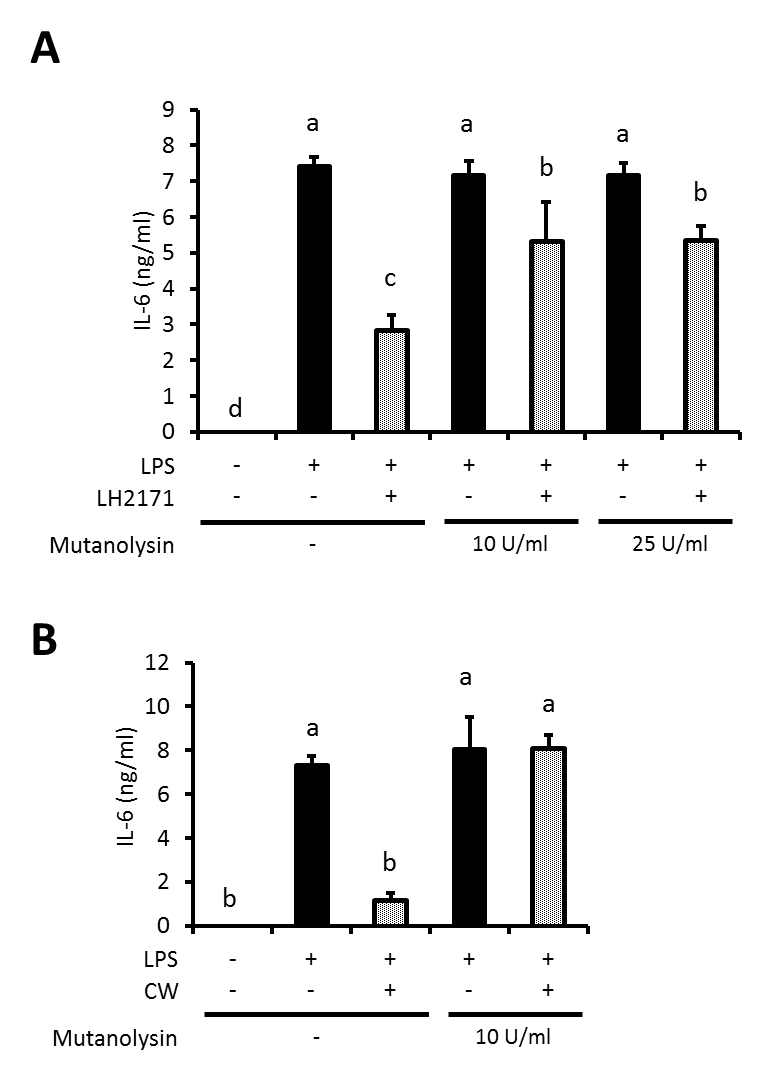


**Supplementary Figure 9**

**Mutanolysin-treatment diminished the inhibitory effect of LH2171 and its cell wall on IL-6 secretion in peritoneal macrophages.**

LH2171 cells or cell wall fractions were treated with different concentration of mutanolysin (0, 10 or 25 U/ml) in buffer (49 mM MES, 1 mM MgCl2, 0.66 mM TES and for 22 hours at 37°C, followed by heat inactivation for 30 min at 80°C. The peritoneal macrophages were pre-incubated with mutanolysin-treated or untreated LH2171 **(A)** or its cell wall **(B)** at a dose of 10 µg/ml for 4 hours and stimulated with 1 µg/ml LPS for 16 hours, after which IL-6 levels in the culture supernatants were determined. Data are shown as mean + SD (n = 3) and analyzed by Tukey-Kramer’s test (^a-d^*P* < 0.05).

**Supplementary Table**

**Supplementary Table 1** Primers for gene expression analysis

| Gene name |  |  | Sequence |  | bp |
| --- | --- | --- | --- | --- | --- |
| *Tnfa* | Forward | 5' | AGCCCACGTCGTAGCAAACCAC | 3' | 22 |
|  | Reverse | 5' | CGGGGCAGCCTTGTCCCTTG | 3' | 20 |
| *Il6* | Forward | 5' | CGTGGAAATGAGAAAAGAGTTGTGC | 3' | 25 |
|  | Reverse | 5' | TGGTACTCCAGAAGACCAGAGGA | 3' | 23 |
| *Il1b* | Forward | 5' | CCCTGCAGCTGGAGAGTGTGGA | 3' | 22 |
|  | Reverse | 5' | TGTGCTCTGCTTGTGAGGTGCTG | 3' | 23 |
| *Nos2* | Forward | 5' | GTTCTCAGCCCAACAATACAAGA | 3' | 23 |
|  | Reverse | 5' | GTGGACGGGTCGATGTCAC | 3' | 19 |
| *Nlrp3* | Forward | 5' | GACCAGCCAGAGTGGAATGAC | 3' | 21 |
|  | Reverse | 5' | AACCTGCTTCTCACATGTCGT | 3' | 21 |
| *Ccl2* | Forward | 5' | CCAGCACCAGCACCAGCCAA | 3' | 20 |
|  | Reverse | 5' | TGGGGCGTTAACTGCATCTGGC | 3' | 22 |
| *Il10* | Forward | 5' | GCCCCAGGCAGAGAAGCATGG | 3' | 21 |
|  | Reverse | 5' | GGGGAGAAATCGATGACAGCGCC | 3' | 23 |
| *Socs1* | Forward | 5' | TCCGATTACCGGCGCATCACG | 3' | 21 |
|  | Reverse | 5' | CTCCAGCAGCTCGAAAAGGCA | 3' | 21 |
| *Tollip* | Forward | 5' | ATTATGGCATGACTCGTATGGAC | 3' | 23 |
|  | Reverse | 5' | CTATACCACTCGTCCTCCACTTG | 3' | 23 |
| *Irakm* | Forward | 5' | TTCCTGGCACGTTCGAATCA | 3' | 20 |
|  | Reverse | 5' | CGCTGCAGCAAAATCCGTTA | 3' | 20 |
| *A20* | Forward | 5' | AAACCAATGGTGATGGAAACTG | 3' | 22 |
|  | Reverse | 5' | GTTGTCCCATTCGTCATTCC | 3' | 20 |
| *Socs3* | Forward | 5' | GGAGATTTCGCTTCGGGACT | 3' | 20 |
|  | Reverse | 5' | CGCTCCAGTAGAATCCGCTC | 3' | 20 |
| *Tlr2* | Forward | 5' | AAGATGTCGTTCAAGGAGGTGCG | 3' | 23 |
|  | Reverse | 5' | ATCCTCTGAGATTTGACGCTTTG | 3' | 23 |
| *Tlr4* | Forward | 5' | CCTGATGACATTCCTTCT | 3' | 18 |
|  | Reverse | 5' | AGCCACCAGATTCTCTAA | 3' | 18 |
| *Rpl19* | Forward | 5' | ATGAGTATGCTCAGGCTACAGA | 3' | 22 |
|  | Reverse | 5' | GCATTGGCGATTTCATTGGTC | 3' | 21 |

**Supplementary Table 2** Antibodies for western blot analysis and fluorescent immunostaining

| Antibodies |  |  | | Source |  |  | Identifier |
| --- | --- | --- | --- | --- | --- | --- | --- |
| Rabbit anti-NF-κB p65 |  |  | | Cell Signaling Technologies | | | Cat#8242 |
| Rabbit anti-Phospho-NF-κB p65 (Ser536) | |  | | Cell Signaling Technologies | | | Cat#3033 |
| Mouse anti-IκBα |  |  | | Cell Signaling Technologies | | | Cat#4814 |
| Rabbit anti-Phospho-JNK (T183/Y185) | |  | | R&D systems | |  | Cat#AF1205 |
| Rabbit anti-JNK |  |  | | Cell Signaling Technologies | | | Cat#9252 |
| Rabbit anti-Phospho-p38 MAPK (Thr180/Tyr182) | | | | Cell Signaling Technologies | | | Cat#9211 |
| Rabbit anti-p38 MAPK |  |  | | Cell Signaling Technologies | | | Cat#9212 |
| Rabbit anti-Erk1/2 |  |  | | Cell Signaling Technologies | | | Cat#9102 |
| Rabbit anti-Phospho-Erk1/2 (Thr202/Tyr204) | | | | Cell Signaling Technologies | | | Cat#9101 |
| Rabbit anti-A20 | | | | Cell Signaling Technologies | | | Cat#5630 |
| Rabbit anti-Socs1 | | | | Cell Signaling Technologies | | | Cat#3950 |
| Rabbit anti-Socs3 | | | | Cell Signaling Technologies | | | Cat#2932 |
| Rabbit anti-β-actin |  | |  | Cell Signaling Technologies | | | Cat#4970 |
| Horse anti-mouse IgG HRP-linked | | |  | Cell Signaling Technologies | | | Cat#7076 |
| Goat anti-rabbit IgG HRP-linked | | |  | Cell Signaling Technologies | | | Cat#7074 |
| Goat anti-rabbit IgG Alexa Fluor® 488-linked | | |  | Abcam | | | Cat#ab150077 |
